# Vitexin induces apoptosis through mitochondrial pathway and PI3K/Akt/mTOR signaling in human non-small cell lung cancer A549 cells

**DOI:** 10.1186/s40659-019-0214-y

**Published:** 2019-02-23

**Authors:** Xiaoli Liu, Qingfeng Jiang, Huaimin Liu, Suxia Luo

**Affiliations:** Department of Integrated Chinese and Western Medicine, Affiliated Cancer Hospital of Zhengzhou University & Henan Cancer Hospital, No. 127 Dongming Road, Zhengzhou City, 450008 Henan Province People’s Republic of China

**Keywords:** Non-small cell lung cancer, Vitexin, Mitochondrial dysfunction, PI3K/Akt/mTOR signaling

## Abstract

**Background:**

Currently, the prognosis of patients with non-small cell lung cancer (NSCLC) remains dismal; hence, it is critical to identify effective anti-NSCLC agents with limited side effects. This study aimed to evaluate the therapeutic potential of flavonoid compound vitexin in human NSCLC cells and the underlying mechanisms.

**Results:**

The experimental results indicated that vitexin reduced the viability of A549 cells in a dose-dependent manner with nearly no toxicity against normal human bronchial epithelial 16HBE cells. Vitexin also dose-dependently increased A549 cell apoptosis, accompanied by the decreased Bcl-2/Bax ratio and the increased expression of cleaved caspase-3. Moreover, the in vivo anticancer activity of vitexin was further determined in nude mice bearing A549 cells. In addition, vitexin induced the release of cytochrome c from the mitochondria to the cytosol and the loss of mitochondrial membrane potential. Vitexin also significantly reduced the levels of p-PI3K, p-Akt and p-mTOR, and the pro-apoptotic effect of vitexin on A549 cells was partly blocked by SC79, an Akt activator.

**Conclusions:**

Accordingly, we believed that vitexin could be used as a potential therapeutic agent for the treatment of NSCLC in the future.

## Introduction

Lung cancer is the leading cause of cancer-related mortality in China [1]. There are two major types of lung cancer: small cell lung cancer (SCLC) and non-small cell lung cancer (NSCLC). NSCLC accounts for approximately 85% of all lung cancer cases [2]. The mechanisms underlying the pathogenesis of NSCLC are complicated. Conventional therapeutic options for NSCLC in clinics include chemotherapy and surgery, but these methods exert limited effects for patients with advanced NSCLC [3]. Undoubtedly, it is of critical importance to identify novel therapeutic agents for the treatment of this fatal malignancy.

**Fig. 1:**
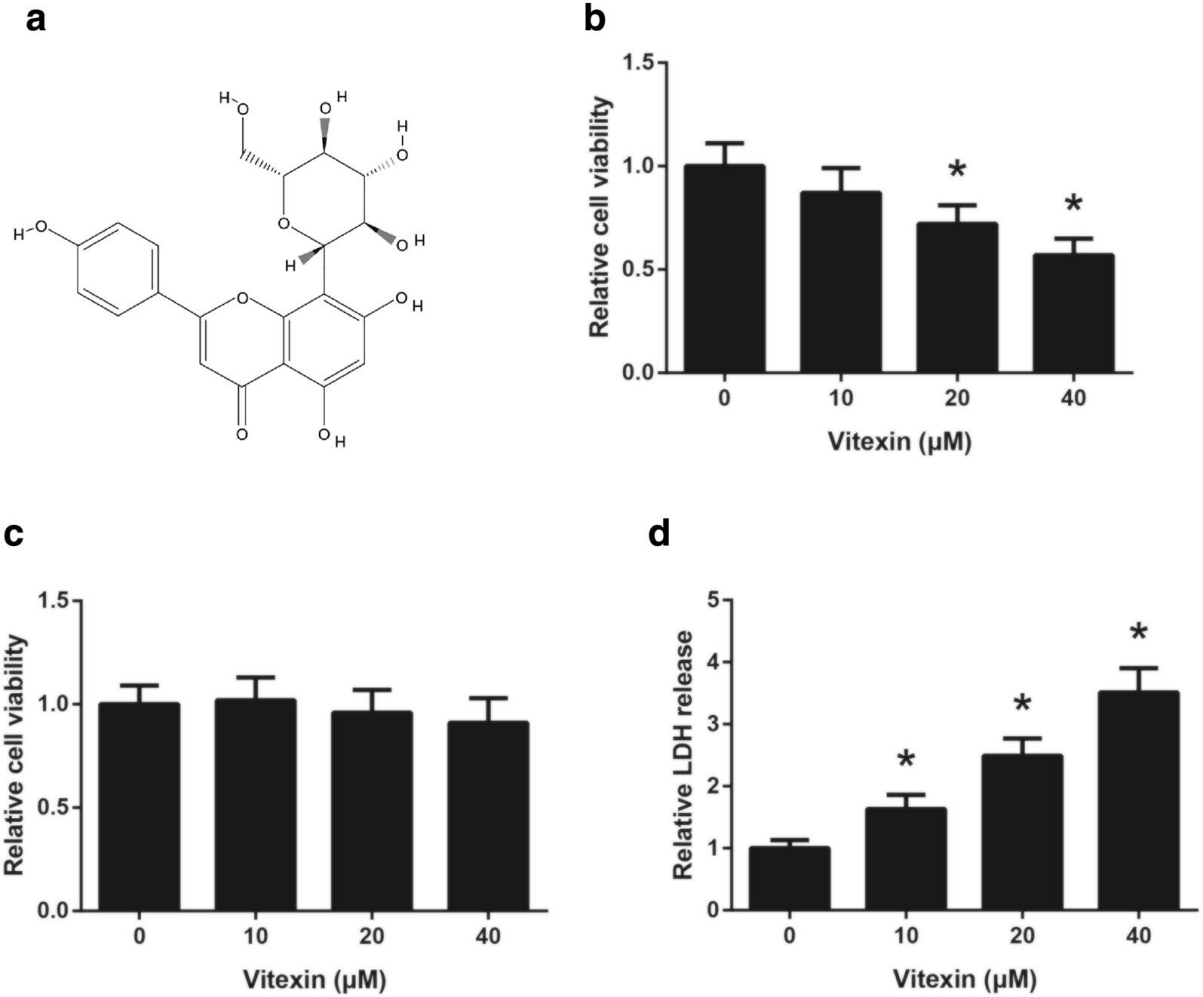
Vitexin reduces viability in A549 cells. **a** The chemical structure of vitexin. **b** The viability of A549 cells following 48 h of vitexin treatment was detected by MTT assay. **c** The viability of 16HBE cells following 48 h of vitexin treatment was detected by MTT assay. **d** The cellular injury of A549 cells following 48 h of vitexin treatment was detected by LDH release assay. *P < 0.05 versus vehicle-treated cells

Recently, natural products, especially plant-derived compounds, have attracted the attention of many researchers for their potential antitumor properties. Among them, vitexin (apigenin-8-C-D-glucopyranoside; Fig. 1a), a naturally-derived flavonoid compound found in the traditional Chinese herb Crataegus pinnatifida (hawthorn) [4], has shown anti-tumor efficacy against a wide variety of human cancers, including leukemia [5], hepatocellular carcinoma [6] and glioblastoma [7]. Therefore, the objectives of the present study were to characterize the anti-NSCLC role of vitexin both in vitro and in vivo, and to clarify the underlying molecular mechanisms.

## Materials and methods

### Cell culture and treatment

NSCLC cell line (A549) and normal human bronchial epithelial cell line (16HBE) were purchased from the Cell Bank of the Chinese Academy of Science (Shanghai, China). These cells were cultured in RPMI 1640 medium (Invitrogen, Carlsbad, CA, USA) containing 10% fetal bovine serum (FBS; Invitrogen), 100 U/ml penicillin and 100 μg/ml streptomycin in a humidified atmosphere of 5% CO_2_ in air.

After attaining 90% confluency in plates, cells were treated with vehicle (0.1% DMSO) or vitexin (Sigma-Aldrich, St. Louis, MO, USA) at doses of 10, 20, and 40 μM for 48 h. To activate Akt, 1 h before vitexin exposure, the cells were pretreated with 5 μM of Akt activator, SC79 (Sigma-Aldrich).

### MTT assay

Cell viability was monitored by 3-(4,5-dimethylthiazol-2-thiazolyl)-2,5-diphenyltetrazolium bromide (MTT) assay. In brief, cells were seeded into 96-well plates at a density of 5 × 10^3^ cells per well. Following treatment with different doses of vitexin for 48 h, 20 μl MTT (5 mg/ml; Sigma-Aldrich) was added to each well, and the cells were incubated for additional 4 h at 37 °C. Formazan cyrstals that formed in living cells was dissolved in 150 μl of DMSO, and the absorbance of the plate was then read with a microplate reader (Dynex, Chantilly, VA, USA) at 490 nm.

### LDH release assay

Cell injury was determined based on lactate dehydrogenase (LDH) leakage into the culture medium from cells using an LDH assay kit (Jiancheng, Nanjing, China) [8]. Following vitexin treatment for 48 h, 100 μl of working solution was added to each well and the plate was incubated for additional 30 min. Then 50 μl stop solution was added to each well, and the absorbance of all samples was detected at 490 nm with a microplate reader.

### Cell apoptosis analysis

Cell apoptosis was determined using an Annexin V-FITC/PI apoptosis detection kit (BestBio, Shanghai, China). In brief, cells were harvested after 48 h of the aforementioned treatment by trypsinization and then double stained with Annexin V-FITC and propidium iodide (PI) for 30 min in the dark. The samples were then analyzed using a FACSCaliber flow cytometer (BD Biosciences).

### Measurement of mitochondrial membrane potential

The mitochondrial membrane potential (MMP) of cells was determined by the classical JC-1 staining method [9]. Briefly, following vitexin treatment, cells were harvested, washed with PBS twice and then incubated with 500 μl JC-1 staining solution (5 μg/ml) for 20 min at 37 °C in darkness. Next, the cells were suspended with trypsin, and analyzed using a flow cytometer.

### Western blot analysis

Total protein was extracted using radioimmunoprecipitation assay (RIPA) lysis buffer (Beyotime, Shanghai, China). For detection of cytochrome c, the mitochondrial and cytosolic fractions were prepared using the Mitochondria/Cytosol Fractionation kit (Abcam, Cambridge, MA, USA). Equal amount of protein for each sample was separated by SDS–polyacrylamide gels and then electro-transferred to polyvinylidene fluoride (PVDF) membranes (Millipore, Bedford, MA, USA). After being blocked in 5% non-fat dried milk in PBS with Tween-20, the membranes were incubated overnight at 4 °C with specific primary antibodies against Bcl-2 (1:1000; Abcam), Bax (1:1000; Abcam), caspase-3 (1:1000; Abcam), cytochrome c (1:1500; Abcam), p-PI3K (1:1000; Cell Signaling Technology, Danvers, MA, USA), PI3K (1:1000; Cell Signaling Technology), p-Akt (1:1000; Cell Signaling Technology), Akt (1:1000; Cell Signaling Technology), p-mTOR (1:1000; Cell Signaling Technology), mTOR (1:1000; Cell Signaling Technology), GAPDH (1:500; Santa Cruz Biotechnology, Inc., Dallas, TX, USA) and COX IV (1:1000; Abcam), followed by incubation with HRP-conjugated secondary antibody at room temperature for 1 h. Then the proteins were detected using an enhanced chemiluminescence kit (Amersham Biosciences, Piscataway, NJ, USA). The results were normalized to GAPDH or COX IV.

### Tumor formation assay

Fifteen male athymic BALB/c nude mice aged 5–6 weeks were purchased from Shanghai Laboratory Animals Center (Shanghai, China) and maintained under SPF conditions. 2 × 10^6^ A549 cells were subcutaneously injected into a single side of the posterior flank of nude mice. Tumor volume was measured using a caliper every 3 days and calculated as follows: Tumor volume (mm^3^) = length × width^2^/2. When the tumor size reached approximately 100 mm^3^, the mice were randomized into three groups (five mice/group). The mice in low dose group and high dose group were treated daily for 4 weeks by intraperitoneal injection with 1 mg/kg and 2 mg/kg vitexin, respectively, and the mice in control group received 0.1% DMSO. At the end of the study (Day 19), the tumors were excised and weighed. All animal handling and procedures were approved by the Ethics Committee of Affiliated Cancer Hospital of Zhengzhou University (Zhengzhou, China). All necessary steps were taken to minimize suffering of the mice.

### Statistical analysis

All statistical analyses were performed using GraphPad Prism 6.0 software (GraphPad Software Inc., La Jolla, CA, USA). All experimental data are shown as the mean ± standard deviation (SD) and analyzed using one-way analysis of variance (ANOVA) and Dunnett’s post hoc test. *P *< 0.05 was considered to indicate a statistically significant difference.

## Results

### Vitexin reduces viability in A549 cells

First, A549 cells were treated with different doses of vitexin, and the inhibitory effect of vitexin on cell viability was estimated by MTT assay. As demonstrated in Fig. 1b, exposure of A549 cells to vitexin for 48 h led to a dose-dependent reduction in cell viability. However, vitexin exerts nearly no toxicity against normal human bronchial epithelial 16HBE cells (Fig. 1c). In addition, we also found that vitexin treatment remarkably increased the LDH leakage of A549 cells (Fig. 1d).

### Vitexin induces apoptosis in A549 cells

**Fig. 2:**
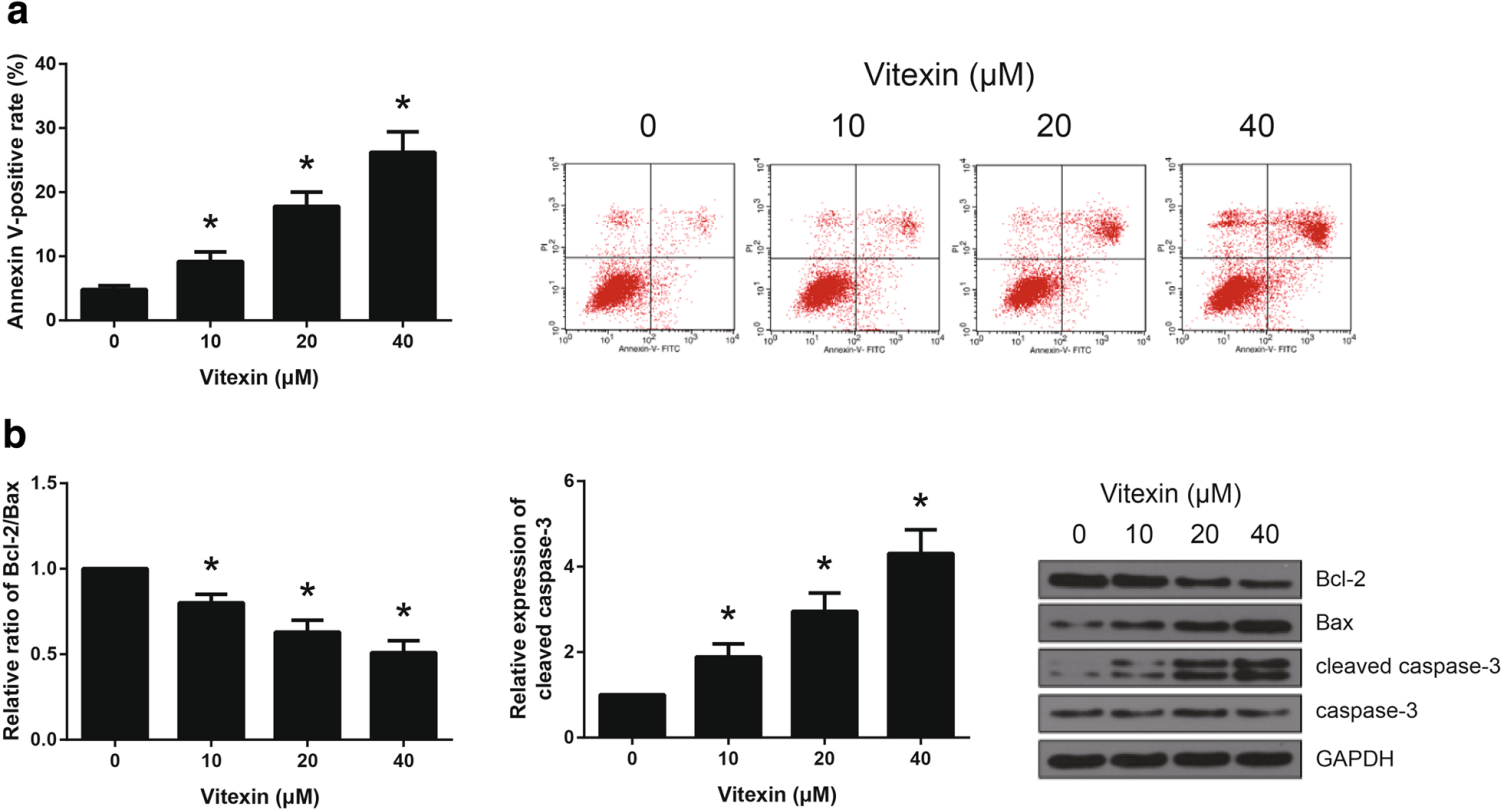
Vitexin induces apoptosis in A549 cells. **a** The apoptosis of A549 cells following 48 h of vitexin treatment was detected by Annexin V-FITC/PI double staining. **b** The expression levels of apoptotic-related proteins in A549 cells following 48 h of vitexin treatment were detected by western blot analysis. *P < 0.05 versus vehicle-treated cells

To determine whether vitexin exert a pro-apoptotic effect on NSCLC cells, flow cytometry analysis via Annexin V/PI staining was performed. As shown in Fig. 2a, vitexin treatment dose-dependently increased the number of Annexin V-positive A549 cells. Next, we investigated the expression levels of several apoptosis-associated proteins by western blot analysis, and the results indicated that vitexin treatment led to the downregultion of Bcl-2/Bax ratio and upregulation of cleaved caspase-3 in A549 cells (Fig. 2b).

### Vitexin inhibits NSCLC tumor growth in vivo

**Fig. 3:**
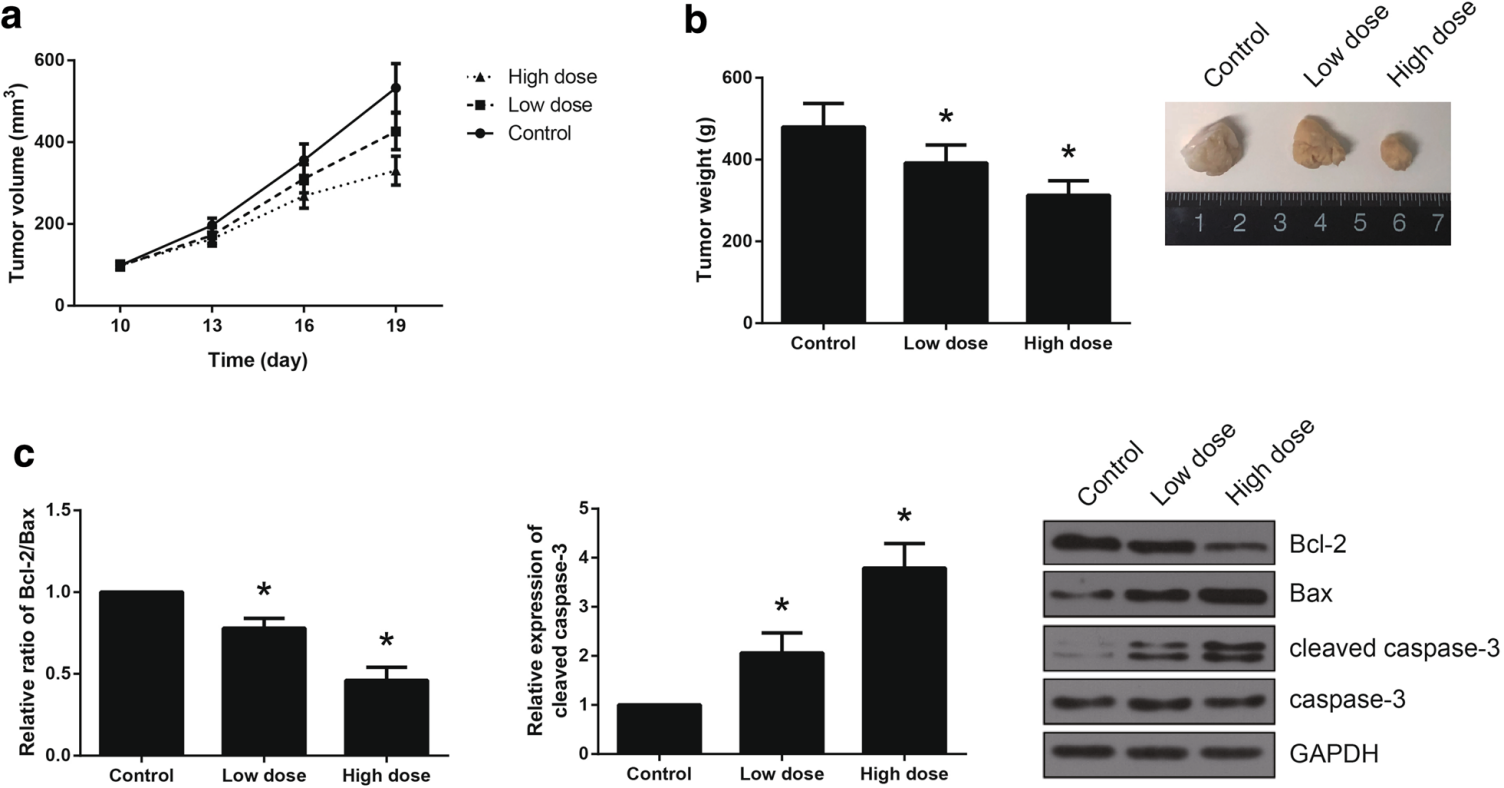
Vitexin inhibits NSCLC tumor growth in vivo. **a** Tumor volume was measured every 3 days and the growth curves were plotted. **b** On Day 19, the tumors were excised and weighted. **c** The expression levels of apoptotic-related proteins in the tumor tissues were detected by western blot analysis. *P < 0.05 versus control group

We also analyzed the anti-NSCLC potential of vitexin in vivo. We confirmed that all mice developed xenograft tumors at the injection sites, and as shown in Fig. 3a, vitexin treatment led to significant inhibition of NSCLC tumor growth. The average weight of tumors was also significantly reduced following vitexin treatment (Fig. 3b). Moreover, we found that Bcl-2 expression was decreased, whereas the expression levels of Bax and cleaved caspase-3 were increased in the tumor tissues of vitexin-treated mice (Fig. 3c).

### Vitexin induces mitochondrial dysfunction in A549 cells

**Fig. 4:**
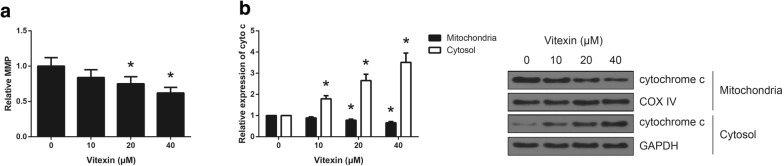
Vitexin induces mitochondrial dysfunction in A549 cells. **a** The loss of MMP in A549 cells following 48 h of vitexin treatment was detected by JC-1 staining. **b** The expression levels of cytochrome c in the mitochondrial and cytosolic fractions of A549 cells following 48 h of vitexin treatment were detected by western blot analysis. *P < 0.05 versus vehicle-treated cells

It is well known that mitochondria play important roles the regulation of cell apoptosis. The results of JC-1 staining indicated that vitexin exposure enhanced the loss of MMP in A549 cells (Fig. 4a). We also observed that vitexin exposure significantly reduced the levels of mitochondrial cytochrome c and increased the levels of cytoplasmic cytochrome c in A549 cells (Fig. 4b).

### Vitexin inactivates PI3K/Akt/mTOR signaling

**Fig. 5:**
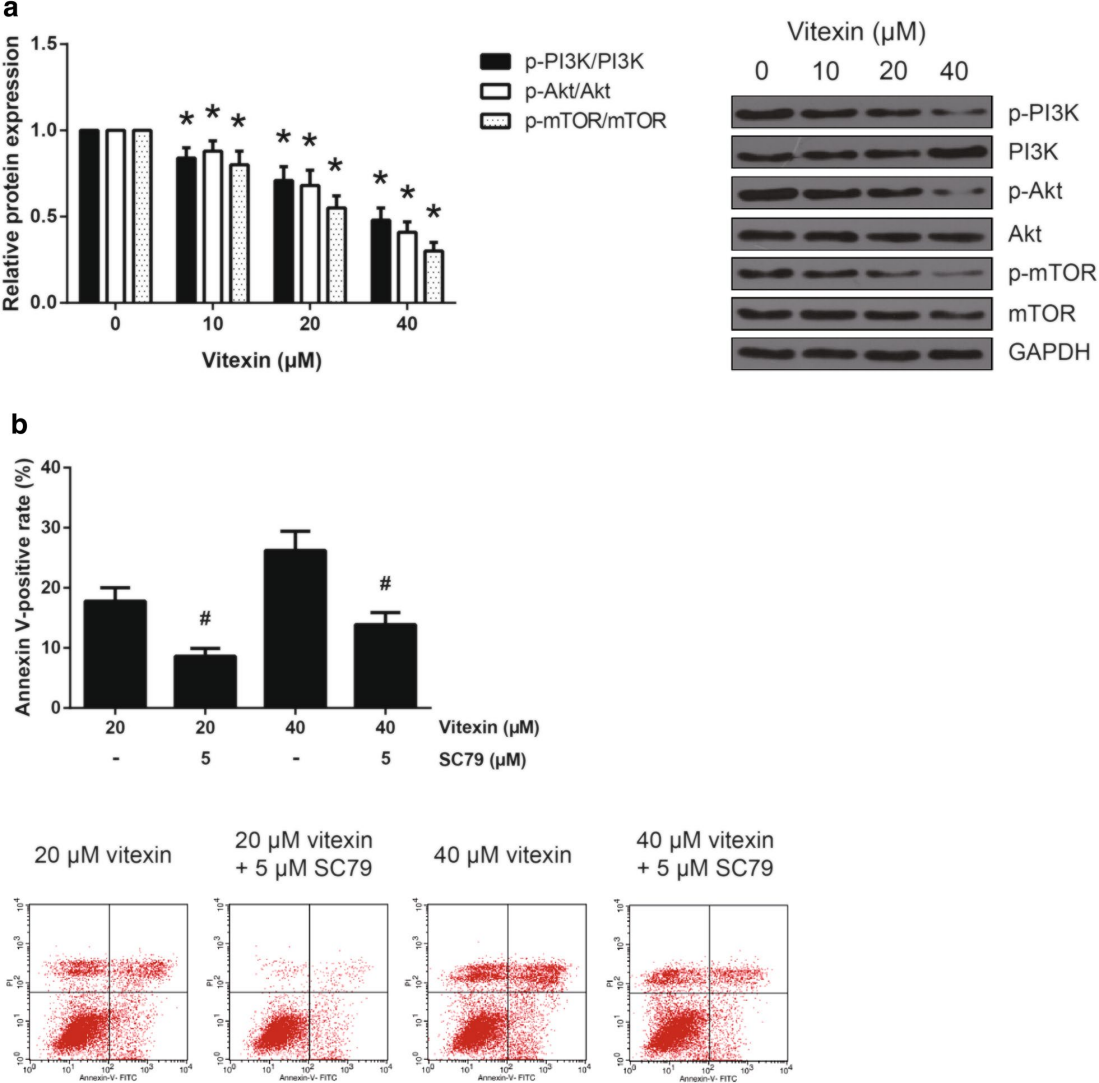
Vitexin inactivates PI3K/Akt/mTOR signaling. **a** The expression levels of PI3K/Akt/mTOR signaling-related proteins in A549 cells following 48 h of vitexin treatment were detected by western blot analysis. **b** The effect of SC79 on the apoptosis of vitexin-treated A549 cells was detected by Annexin V-FITC/PI double staining. *P < 0.05 versus vehicle-treated cells; ^#^*P *< 0.05 versus SC79-untreated cells

Targeting PI3K/Akt/mTOR signaling is a promising approach for the treatment of NSCLC [10]. We further investigated the effect of vitexin on PI3K/Akt/mTOR signaling in NSCLC cells. The results of western blot analysis demonstrated that vitexin treatment dose-dependently reduced the levels of p-PI3K, p-Akt and p-mTOR in A549 cells (Fig. 5a). Additionally, as demonstrated in Fig. 5b, the apoptosis-inducing role of vitexin in A549 cells was also significantly blocked by pretreatment with 5 μM of Akt activator, SC79.

## Discussion

Currently, chemotherapy remains the major therapeutic option for NSCLC patients [11]; however, anticancer agents often have harmful side effects. Major progress has been made in identifying novel anti-NSCLC agents with low toxicity. Vitexin possesses potential antitumor activities against many human cancers. For example, vitexin could inhibit esophageal cancer cell growth and induce apoptosis [12]. In the present study, we found that vitexin treatment reduced the viability of A549 cells in vitro, accompanied by an increase in LDH release due to cell membrane damage. In addition, administration of vitexin also inhibited the NSCLC tumor growth in vivo. Hence, the anti-NSCLC potential of vitexin was clearly indicated.

Apoptosis is an evolutionary conserved program of cell death, and activation of apoptotic pathways is an important anti-cancer strategy [13]. The Bcl-2 family proteins, including pro-apoptotic Bax and anti-apoptotic Bcl-2, play important roles in the regulation of apoptosis and tumorigenesis [14]. Mitochondrion is an important organelle involved in cell death [15], and loss of MMP can induce the release of pro-apoptotic molecules. Our findings demonstrated that vitexin reduced the Bcl-2/Bax ratio and caused the release of cytochrome c from mitochondria to cytosol, which further led to the cleavage of caspase-3, an executor caspase, in A549 cells. Therefore, we considered that vitexin induces A549 cell apoptosis, in part, through mitochondria-dependent pathway.

The PI3K/AKT/mTOR signaling is one of the most important intracellular pathways, which serve a critical regulatory role in a number of key cancerous behaviors [16, 17]. Over-activation of this signaling is observed in NSCLC and many others. Drugs that target PI3K/Akt/mTOR signaling have the potential to induce apoptosis in NSCLC cells [18]. In this study, we observed that treatment of A549 cells with vitexin reduced the levels of p-PI3K, p-Akt and p-mTOR, and more importantly, pretreatment with Akt activator, SC79, effectively blocked vitexin-induced A549 cell apoptosis. These results suggested that vitexin induces apoptosis partly through suppressing PI3K/Akt/mTOR signaling in A549 cells.

## Conclusion

Taken together, by performing in vitro and in vivo experiments, our study might be the first to show that vitexin treatment impairs the viability and induces the apoptosis of A549 cells partly through mitochondrial pathway and PI3K/Akt/mTOR signaling. Although more experiments are required, based on the findings of our study, vitexin might be an innovatively effective agent in the treatment of NSCLC.

## Abbreviations

NSCLC: non-small cell lung cancer
SCLC: small cell lung cancer
FBS: fetal bovine serum
LDH: lactate dehydrogenase
PI: propidium iodide
MMP: mitochondrial membrane potential
RIPA: radioimmunoprecipitation assay
PVDF: polyvinylidene fluoride
SD: standard deviation
